# Extraction, integration and analysis of alternative splicing and protein structure distributed information

**DOI:** 10.1186/1471-2105-10-S12-S15

**Published:** 2009-10-15

**Authors:** Matteo D'Antonio, Marco Masseroli

**Affiliations:** 1 IFOM-IEO Campus, Via Adamello 16, Milan, 20139, Italy; 2 Dipartimento di Elettronica e Informazione, Politecnico di Milano, Piazza Leonardo da Vinci 32, Milan, 20133, Italy

## Abstract

**Background:**

Alternative splicing has been demonstrated to affect most of human genes; different isoforms from the same gene encode for proteins which differ for a limited number of residues, thus yielding similar structures. This suggests possible correlations between alternative splicing and protein structure. In order to support the investigation of such relationships, we have developed the Alternative Splicing and Protein Structure Scrutinizer (PASS), a Web application to automatically extract, integrate and analyze human alternative splicing and protein structure data sparsely available in the Alternative Splicing Database, Ensembl databank and Protein Data Bank. Primary data from these databases have been integrated and analyzed using the Protein Identifier Cross-Reference, BLAST, CLUSTALW and FeatureMap3D software tools.

**Results:**

A database has been developed to store the considered primary data and the results from their analysis; a system of Perl scripts has been implemented to automatically create and update the database and analyze the integrated data; a Web interface has been implemented to make the analyses easily accessible; a database has been created to manage user accesses to the PASS Web application and store user's data and searches.

**Conclusion:**

PASS automatically integrates data from the Alternative Splicing Database with protein structure data from the Protein Data Bank. Additionally, it comprehensively analyzes the integrated data with publicly available well-known bioinformatics tools in order to generate structural information of isoform pairs. Further analysis of such valuable information might reveal interesting relationships between alternative splicing and protein structure differences, which may be significantly associated with different functions.

## Background

Most of the genes in higher eukaryotes contain introns. The presence of many introns in higher eukaryotic genes allows the expression of different proteins (isoforms) in different tissues from a single gene, phenomenon known as alternative splicing. In lower eukaryotes, however, alternative splicing is very rare [1].

It has been estimated that at least 60% of human genes produces alternatively spliced forms [2], but only for a small part of the human genes the alternative splicing variants have been detected, because the regulatory processes which lead to alternative splicing have not been well understood yet. Alternative splicing may be important in understanding cancer, since some cancer-associated genes have alternatively spliced forms which differ from the forms in normal tissues. Generally, aberrant splicing events are responsible for pathologies [3].

Alternative splicing is important also from an evolutionary point of view: different transcripts may be translated in different departments of the cell, in different tissues and at different developmental stages, giving rise to cell, tissue and developmental diversity and specificity; alternative splicing also allows evolution of the gene structure [4]. It has been demonstrated that the alternative first exon plays an important role in protein diversity at cell and tissue level [5,6].

**Figure 1:**
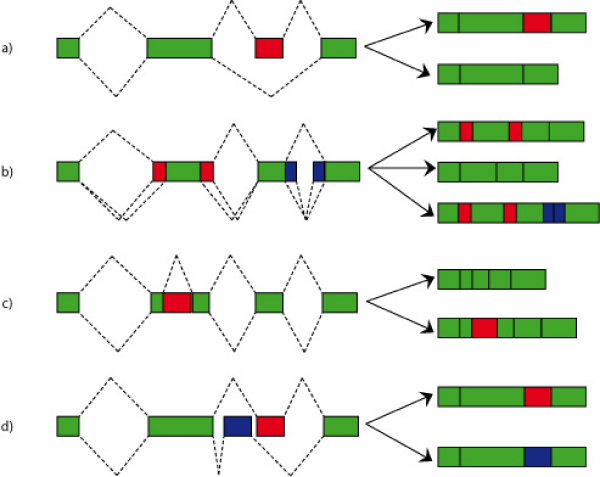
**Events of alternative splicing**. In a typical alternatively spliced gene, where most of the exons are constitutive (i.e. they are always included in the final mRNA), four different types of alternative splicing events may occur to give rise to different final transcripts: a) cassette exons (one exon is skipped in some transcripts), b) isoforms of introns or exons (their boundaries may be different in different transcripts, with consequent truncation/extension of the flanking introns/exons), c) intron retentions (one intron is not spliced out and may be inserted into the final transcript), d) mutually exclusive exons (different exons may be included in different final transcripts). On the right of the figure the alternative mRNA transcripts are displayed; conserved parts are colored in green, while alternative elements are colored in red or blue.

In a typical alternatively spliced gene most of the exons are constitutive; hence they are always included in the final mRNA transcript. The alternative part is composed by:

- skipped exons (cassette exons and mutually exclusive exons): an entire exon appears in some transcripts but not in others;

- exon/intron isoforms: the borders of the exon/intron are different, leading to truncation/extension of introns/exons;

- intron retentions: an intron is not spliced out (Figure 1).

In addition to these, alternative promoters or alternative poly-A tails exist: in this case the coding sequence is the same for all the isoforms, but the untranslated regions may vary, e.g. alternative promoters that involve different transcriptional controls.

There are often functional differences among spliced forms: insertion, deletion or substitution of protein domains by alternative splicing frequently modifies protein function by inserting or deleting functional residues or by substituting the sequence that includes a functional site. Alternative splicing inserts or deletes whole protein domains; it does not occur within protein domains. This can be explained by the fact that exons may reflect domain boundaries, or that natural selection may eliminate meaningless alternative splicing variants which do not result from full domains [7].

To investigate how alternative splicing affects the protein function, it is important to understand whether the protein structure is influenced by the insertion/deletion/substitution of residues. The secondary structure of a protein is determined when special rearrangements result from the folding of localized parts of a protein. Many types of different secondary structures may be present inside the same protein, due to different non-covalent interactions between the residues, e.g. hydrogen bonds produce to alpha helix and beta sheet secondary structures. Averagely, inside a protein 60% of the chain is alpha helix or beta sheet, the other 40% is loop. To date, many different alphabets to define the secondary structure exist; the most commonly used alphabet is the Definition of Secondary Structure of Proteins (DSSP) [8], which includes 8 types of structures: *H *(alpha helix), *B *(isolated beta-bridge), *E *(extended strand, participating in beta ladder), *G *(3-helix, i.e. 3/10 helix), *I *(5-helix, i.e. pi helix), *T *(hydrogen bonded turn), *S *(bend), and (other).

When analyzing the protein structure, two additional properties are worth to be investigated: accessibility and flexibility. The former is a measure of the static solvent exposure, i.e. the number of water molecules which are in contact with every residue of the protein: the average accessibility of the protein is calculated as the average value over all the residues. The latter, which refers to the vibration of an atom around its rest position, is measured as thermal motion of the alpha carbon of every residue [8].

To automatically extract, integrate, and analyze the alternative splicing and protein structure data sparsely available in different distributed databanks, we created a Web application called Alternative Splicing and Protein Structure Scrutinizer (PASS). In order to build the PASS Web application, we: 1) defined a database schema suitable to store and integrate alternative splicing and protein structure information extracted from the Alternative Splicing Database (ASD) [9], the Ensembl databank [10], and the Protein Data Bank (PDB, ) [11]; 2) developed a software capable of creating and updating the database by automatically retrieving and integrating data from different databanks accessible through the Internet; 3) created a software to analyze the retrieved data and store the results from the analysis inside the database, and 4) designed and implemented a Web interface that allows users to query the database in order to examine the integrated data and use them to evaluate their own gene or protein sets.

## Results and discussion

### PASS database and designed analysis steps

**Figure 2:**
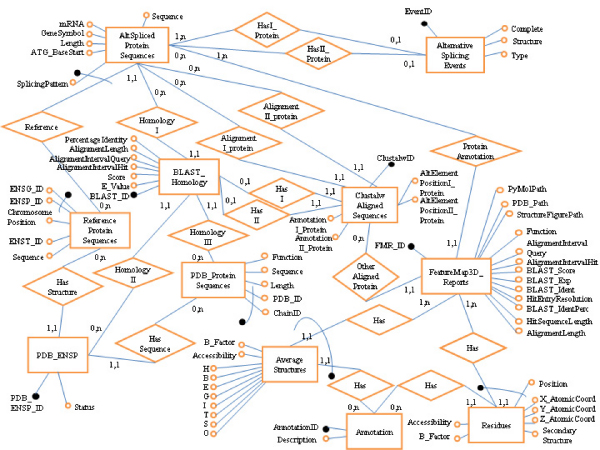
**Entities-Relationships diagram of the PASS database**. The PASS database is composed of several parts: *ReferenceProteinSequences*, *AltSplicedProteinSequences*, *AlternativeSplicingEvents *and *PDB_ProteinSequences *contain the primary data from ASD, Ensembl and PDB; *PDB_ENSP*, *BLAST_Homology *and *ClustalwAlignedSequences *contain the analysis data from PICR, BLAST and CLUSTALW, respectively; *Annotation *contains the different possible positions defined for the alternative elements of a couple of protein sequences (and used in the residue annotation); while *FeatureMap3D_Reports*, *AverageStructures *and *Residues *contain the FeatureMap3D analysis results and their processing.

The relational PASS database has been specifically designed and built by using MySQL DBMS in order to integrate and store the alternative splicing and protein structure primary data gathered from three publicly available databanks (ASD, Ensembl and PDB), as well as the results of their analysis. It includes data about: 1) the alternatively spliced protein sequences (e.g. Ensembl identifier for the codifying gene, splicing pattern, and peptide sequence); 2) the alternative splicing events (e.g. type of alternative splicing, splicing patterns among which it takes place, and the introns/exons on which the alternative splicing occurs); 3) the reference protein sequences defined by Ensembl (e.g. Ensembl identifier for the protein and the codifying gene, cytogenetic location, and peptide sequence); 4) the protein structures (e.g. identifiers and sequences from PDB). All these data are imported and respectively stored in the *AltSplicedProteinSequences*, *AlternativeSplicingEvents*, *ReferenceProteinSequences*, and *PDB_ProteinSequences *tables of the PASS database (Figure 2).

To obtain information about the possible relationship between alternative splicing and protein structure, a multi-step analysis procedure of the integrated data has been designed. The first step concerns the filtering of all the reference protein sequences in Ensembl in order to consider only those proteins which have a resolved structure in PDB; this allows performing all the subsequent analyses on a smaller subset of proteins, thus limiting the computational load required, in particular, by some time-consuming operations. The filtering is achieved by using the Protein Identifier Cross-Reference (PICR, ) Web service, which provides a mapping between Ensembl and PDB identifiers [12]. PICR maps 3,480 Ensembl human genes, which correspond to 15.1% of the total human genes in Ensembl, to 1,942 distinct PDB structures, with a total of 13,972 associations (4 structures average for each gene, as more than one protein structure in PDB can be associated to the same alternative splicing gene). Mapping results are stored in the *PDB_ENSP *table of the PASS database (Figure 2).

A further filtering step is made by performing BLAST [13] alignments against PDB of each isoform sequence selected through PICR, in order to assess the associations between ASD isoforms and PDB structures suggested by the correspondences between Ensembl reference sequences and PDB structures obtained from PICR. This BLAST search selects 3,056 best alignments between isoforms from ASD and PDB structures (9.5% of the isoforms in ASD), which correspond to 953 genes (9.6% of the genes in ASD, and 4.1% of the human genes in Ensembl). BLAST results are stored in the *BLAST_Homology *table of the PASS database (Figure 2).

Once all proteins with a resolved structure are selected, CLUSTALW [14] is executed on each couple of isoforms for which an alternative splicing event is defined in ASD (i.e. imported and stored in the *AlternativeSplicingEvents *table of the PASS database). This allows annotating each residue of the two isoforms according to the alignment between their alternatively spliced sequences: hence it is possible to verify whether a residue is conserved between the two spliced forms, and whether there are mismatches or gaps. Through CLUSTALW the annotation for both aligned sequences and the position of the alternative elements are determined. Seven different possible positions for the alternative elements were defined and stored in the *Annotation *table of the PASS database (Figure 2): *0 *(inner alternative element), *1 *(more than one alternative element), *2 *(terminal alternative element), *3 *(only mismatches between the two sequences are present), *4 *(element aligned to a gap: this happens if the two sequences do not overlap), *5 *(element shared between the two sequences), *6 *(either mismatches or insertions are defined). The data derived from the analysis with CLUSTALW are stored in the *ClustalwAlignedSequences *table of the PASS database (Figure 2). In ASD 52,248 events of alternative splicing are described for 23,295 isoforms (72.6% of the isoforms defined in ASD) and 6,384 genes (64.2% of the genes in ASD). By combining the alternative splicing event data with the results from the previous step of BLAST filtering and the residue annotation through CLUSTALW, 3,951 alternative splicing events are annotated (7.6% of the events in ASD), which regard 599 genes and 2,149 isoforms (6% of the genes with alternative splicing, and 6.7% of the total isoforms in ASD).

The last step was designed in order to determine in which type of protein secondary structure each annotated residue of alternatively spiced isoforms is involved. Data from the previous annotation step are processed using FeatureMap3D [15], which uses BLAST to align a query sequence to PDB entries, thus predicting the structure of the input sequence. BLAST results from FeatureMap3D are stored in the *FeatureMap3D_Reports *table of the PASS database (Figure 2). FeatureMap3D also provides a detailed report about the protein secondary structure in which every single residue in the query sequence, which is aligned against its best match sequence in PDB, is involved. Data from these reports are stored in the *Residues *and *AverageStructures *tables of the PASS database (Figure 2). The former table includes all the structural data, residue by residue (position on the sequence, coordinates, secondary structure, annotation from CLUSTALW, accessibility and flexibility), while the latter includes the average structural distribution, both for the whole protein and divided by type of annotation. Thus, these data define whether a particular structural pattern is present for a particular type of alternative splicing event. By processing the sequences from the annotations generated by CLUSTALW, FeatureMap3D defines 5,604 structures to which 768,477 residues of 1,569 isoforms coming from 480 genes (4.8% of the genes with alternative splicing) belong. Table 1 shows a comprehensive view of the amount of data stored in the PASS database.

**Table 1:** Summary of the data integrated and stored in the PASS database

**Source and type of data**		**Total**	**% of total in Ensembl**	**% of total in ASD**
**Ensembl**	**genes**	22,997	100.00	-
	**isoforms**	46,951	100.00	-
**ASD**	**genes**	9,945	43.24	100.00
	**isoforms**	32,079	68.32	100.00
	**alternative splicing events**	52,248	-	-
**PDB**	**structures**	111,015	-	-
**Ensembl genes with a resolved structure (from PICR)**		3,480	15.13	-
**Mapping from BLAST against PDB**	**genes**	953	4.14	9.58
	**isoforms**	3,056	6.51	9.53
**Alignments produced by CLUSTALW**	**genes**	599	2.60	6.02
	**isoforms**	2,149	4.58	6.70
**Results from FeatureMap3D**	**genes**	480	2.09	4.83
	**isoforms**	1,569	3.34	4.89
	**residues**	768,477	-	-
	**structures**	5,604	-	-

### Software system to create and update the database automatically

**Figure 3:**
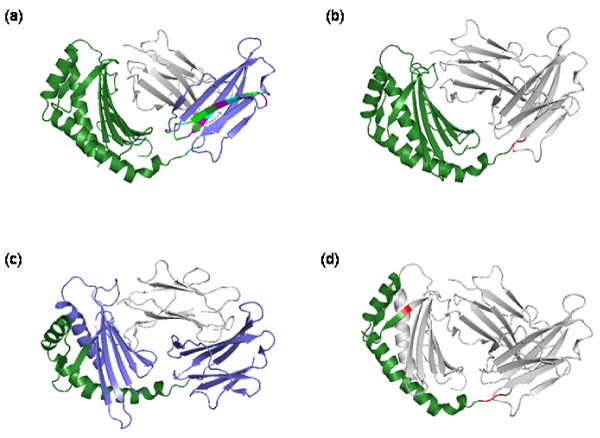
**Protein structures colored according to their residue annotation**. Example of the isoforms for the gene ENSG00000104870 (IgG receptor FcRn large subunit p51 precursor): the same isoform (a, c) is aligned using CLUSTALW to two other different isoforms (b, d) of the same gene. The parts conserved between the two aligned isoforms are colored in green, the residues conserved only in one isoform are colored in blue (the first and last residue of the insertion are colored in red in the other isoform), and the different mismatches between the two sequences are depicted in other colors, based on whether the substitution has a positive or negative value in the BLOSUM62 matrix. The images are obtained with PyMOL .

All steps of the procedure previously described are executed automatically through a system of Perl scripts specifically implemented. The first step consists in the download, with consequent extraction and import into the PASS database, of the primary data from ASD, Ensembl and PDB databanks. After populating the corresponding tables inside the database, the following steps are performed by automatically executing the PICR, BLAST and CLUSTALW software and storing the results of each step in the PASS database. The results from CLUSTALW are also saved into a text file required as input to FeatureMap3D software, which is executed online  to complete the last step of the analysis. Results from FeatureMap3D are stored in 4 distinct files for each considered protein sequence: the PDB file of the protein structure, a PyMOL script (generated automatically by FeatureMap3D) to color the structure according to the considered sequence annotation (Figure 3), a PNG image of the structure, and a report file with the description of the protein secondary structure residue by residue. This file is parsed and its content is stored into the *Residues *and *AverageStructures *tables in the PASS database.

### PassUsers database

In order to manage users' registrations to the PASS Web application and users' input and PASS database search data, the relational PassUsers database has been developed by using MySQL DBMS. It allows: 1) maintaining the information about PASS registered users and recognize them when they log in; 2) storing all the data the registered users upload in the PASS system; 3) storing all the queries to search and extract data in the PASS database that the registered users manually define and want to save in order to be performed at later time without needing to redefine them.

**Figure 4:**
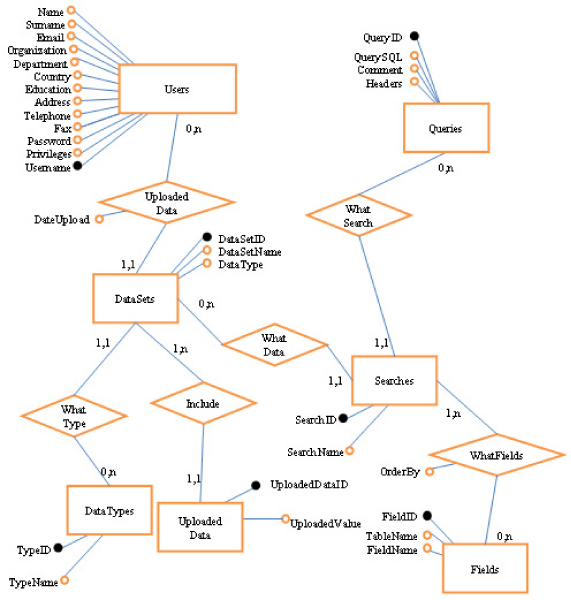
**Entities-Relationships diagram of the PassUsers database**. The PassUsers database is composed of three main parts that store: the registration data for every user (*Users*), the data uploaded by the user (*DataSets*, with the definition of the uploaded datasets; *DataTypes*, with the definition of the type of data uploaded; and *UploadedData*, with all the identifiers uploaded for every dataset), and the user's search data (*Searches*, with the searches defined by every user; *Queries*, with all the queries the user may make; and *Fields*, with the information about what field in the SELECT statement of the query the user has chosen to display).

The PassUser database is composed of several tables. The *Users *table contains the data requested for the PASS user registration (Figure 4). The registered user may upload his or her own set of data in the PASS system, where they are stored in two tables: *DataSets*, which includes the information of the user's uploaded datasets, and *UploadedData*, which contains all the uploaded data. The *DataTypes *table defines whether the data uploaded by the user are composed of ENSP identifiers, ENSG identifiers or types of alternative splicing events (e.g. "cassette exons").

The *Queries *table contains all the predefined SQL queries the user can use to interrogate the PASS database. Each record of the table contains information about the FROM and WHERE clauses of the SQL queries, while the SELECT clauses of the queries are visually composed by the user and stored in the *Fields *table associated to the user performed database searches stored in the *Searches *table. In this way, the user can choose the query to perform, as well as the result fields to display in the Web interface, and can save them to be used at later time to perform the desired searches in the PASS database.

### Web interface

**Figure 5:**
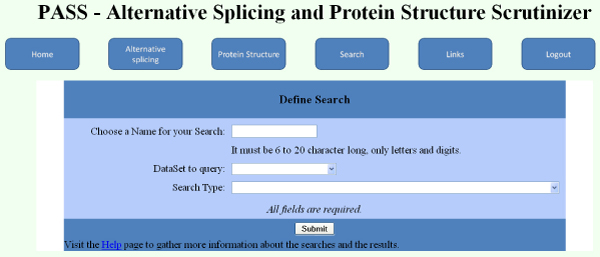
**Search section of the PASS Web application**. Screenshot of the PASS search main page that enables users to define the initial parameters of a search to be performed in the PASS database.

By using Javascript and Active Server Pages scripts, a Web interface has been implemented to enable PASS users to easily extract and further analyze all the data stored in the PASS database, and use them to evaluate their own gene or protein datasets. Two types of users have been defined: basic and privileged, which can also save their searches and access them at later time. Privileged status is granted by PASS administrators upon evaluation of user request. After registering to PASS, users can upload their Ensembl gene or protein identifiers, or splicing event types, as defined in the ASD database, and perform several kinds of searches, including some that allow alternative splicing and structural data analysis of their uploaded genes and proteins. In the search main Web page (Figure 5) the user can define a name for the search to perform (so that it can be saved and performed again at later time) and select the user's uploaded dataset (of gene, protein or splicing event identifiers) to use for searching information in the PASS database, and the query to apply. In the subsequent Web page, the user can also define what specific data field (among all those extractable with the chosen query) to display, and in which format (table or bar plot) to display or download the results.

**Figure 6:**
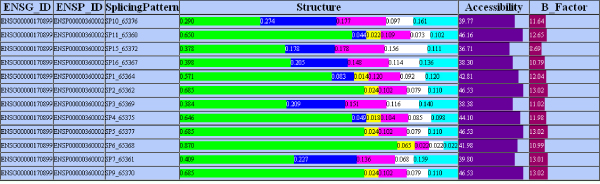
**Example of structural analysis**. Isoforms (identified by the ENSP code of the reference protein and their splicing pattern) of the gene ENSG00000170899 (Glutathione S-transferase A4) and bar plot composition of their structure: alpha helices (green), extended strands participating in beta ladder (blue), 3-helices (yellow), hydrogen bonded turns (purple), bends (white), and other structures (cyan); the last two columns on the right contain the bar plots of the accessibility (static solvent exposure) and flexibility (B factor) values of the isoforms.

Several queries have been implemented for the PASS users. The simplest of them allows the user to extract in tabular format information about alternatively spliced protein sequences, alternative splicing events, proteins with a resolved structure, or protein annotations from CLUSTALW. A second set of queries, which regards the data from the structural analysis, allows performing different investigations based on the results from FeatureMap3D. First, a user can gather information about the average secondary structure of the alternatively spliced protein sequences referenced in a dataset; in this case, the user may choose to display query results: 1) in a simple table with a column for each data field, or 2) as bar plots of the secondary structure distributions, together with bar plots of accessibility and flexibility values for the considered proteins (Figure 6), in order to visually detect different patterns of secondary structure distribution for the different isoforms. Then, similarly, the user may extract the secondary structure distribution for the different types of annotation (by type of alternative splicing event, or by alternative element position), in order to detect whether insertions/deletions/mismatches bring variations in the protein structure. These analyses may be of fundamental support for several investigations:

- to determine whether a particular type of insertion has a preferred structural pattern;

- in presence of an inner alternative exon, to evaluate whether the inserted element might be more rigid or more flexible than the rest of the protein;

- in presence of mismatches, to determine whether the substituted residues are on the protein's surface (as indicated by high accessibility values); furthermore, if these have a low similarity with those in the other splicing pattern (as indicated by their low values in the BLOSUM62 matrix [16]), then the overall properties of the isoforms, such as polarity and solubility, might differ significantly.

A last set of implemented queries allows the PASS user to export the processed data stored in the PASS database by downloading query results as flat files, which may be inputted to other programs such as Excel, MatLAB or R, in order to further analyze them. Towards this aim, several possibilities have been implemented: 1) extract the average structure and the accessibility and flexibility values for all the alternatively spliced genes; 2) extract the average structures sorted by annotation, in order to determine whether conserved residues have a different structural pattern than the alternatively spliced ones; 3) extract the average structures sorted by position of the alternative element, in order to understand whether a particular type of insertion may affect the protein structure; and 4) extract the average structures sorted by the type of alternative splicing event, in order to understand whether the event type may affect the protein structure.

## Conclusion

The Alternative Splicing and Protein Structure Scrutinizer (PASS) has been developed as a Web application able to make large scale automatic analyses of alternative splicing and protein structures of human genes. PASS can provide numerous results: from the simple analyses of protein sequences (either structural or about alternative splicing), to the extraction of protein structural information, which is the basis for determining the relationship between alternative splicing and protein structure. All data processing and analyses are performed automatically; this allows executing large scale analyses: more than one thousand protein structures have been investigated and results are available in the PASS database. By using them the PASS Web application is able to process all the human genes and proteins required by the user and provide information about the analysis of their structure. The results may be either visual (in form of bar plots that enable the user to immediately perceive the differences in protein secondary structure distribution among the considered sequences), or in form of tables that may be downloaded for further investigations with specific software tools. To our knowledge, at present there is no other software or databank available that can provide similar integrated information. Evaluation of such valuable information stored in the PASS database might reveal interesting correlations between alternative splicing and protein structure differences significantly associated with different functions.

## Methods

### Data sources

**Figure 7:**
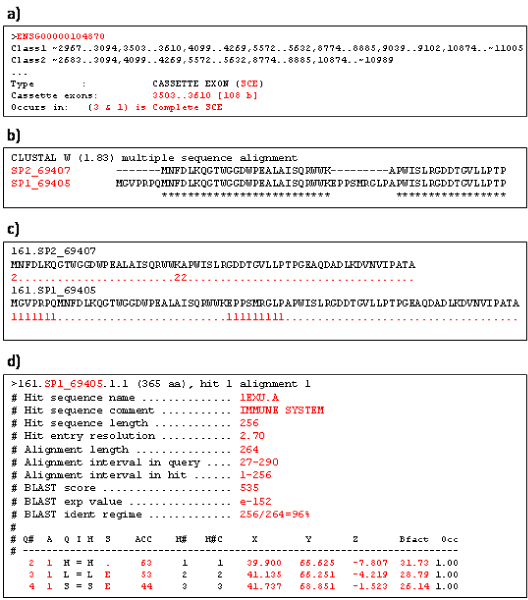
**Examples of the primary data considered**. a) Example data of the alternative splicing events of gene ENSG00000104870 extracted from the ASD database. b) Output alignment from CLUSTALW of two alternatively spliced proteins from the gene in a); * indicates a conserved residue. c) Residue annotation defined for the pair of CLUSTALW aligned sequences in b); the residues labeled as "1" are present only in one sequence, the residues labeled as "2" represent the positions where residues are inserted in the other sequence, and the residues labeled as "." are conserved in the two sequences. d) Output report of the FeatureMap3D analysis of the annotated sequences displayed in c); the data colored in red are those extracted and stored into the PASS database.

As source of information we considered the following three databanks:

- The Alternative Splicing Database (ASD) from the European Bioinformatics Institute (EBI) [9] was used as source for human alternative splicing data. This is a computer generated high quality databank which includes data from gene/transcript sequence (EST/mRNA) alignments. These data are cleaned from ambiguities and analyzed: alignment gaps are potential introns while matches in the alignment correspond to exons if they are flanked on both sides by introns. Introns and exons which overlap with one another correspond to alternative splicing events; these events are described as skipped exons, exon/intron isoforms, or alternative exon events. The alternative splicing events are defined between different isoforms from a single gene: each of them contains the type of alternative splicing, the splicing patterns between which the event occurs, and the introns/exons which are involved in the alternative splicing event. The latest version (release 3) of the alternatively spliced protein sequences (*AltSplice-rel3.peptides.fasta*) and the definition of the alternative splicing events (*AltSplice-rel3.events.txt*) have been downloaded from the ASD FTP site at EBI . The ASD includes analyses on 9,945 genes and 32,079 isoforms, among which 52,248 events of alternative splicing are defined (Figure 7a).

- The Ensembl databank [10] was used as reference benchmark for the human genes. The release 48 of the list of human reference protein sequences, which includes 22,997 human genes, was downloaded from the Ensembl FTP site . Each considered entry contains the Ensembl gene and protein identifiers, the position of the gene on the chromosome, and the protein sequence.

- The Protein Data Bank (PDB) [11] release of February 10^th ^2008 with protein sequences in FASTA format (*pdb_seqres.txt*) has been downloaded from ; 111,015 protein structures are included in this release.

### Bioinformatics software and tools

Within the PASS Web application the bioinformatics tools following described are used:

- The Protein Identifier Cross-Reference (PICR) [12] has been developed by EBI to map identifiers and sequences between different databanks. We used its Web service version to map all the Ensembl protein identifiers (ENSP) to the PDB identifiers, in order to avoid doing a BLAST of all Ensembl against all PDB.

- BLASTALL, which has been downloaded as stand alone executable program from , is used to blast alternatively spliced protein sequences against PDB; it has been chosen because it allows defining the database of hit sequences to be used. To this aim, two steps need to be undertaken: 1) define the database of hit sequences: this is done by running the command "*FORMATDB"*, and 2) run the command "*BLASTALL -p BLASTP -m 8"*, where the parameter "*-p BLASTP*" defines that the BLAST is made for amino acid residues and the parameter "*-m 8*" defines that the output must be in tabular format [13]. By taking advantage of the results from PICR, only the alternatively spliced protein sequences from the genes which have a significant hit in PDB are aligned against the protein sequences which have a defined three dimensional structure in PDB. Proceeding this way, the computational load is significantly reduced in comparison with an all-against-all BLAST search.

- CLUSTALW [14], downloaded as stand alone executable program from [14], is used to annotate every residue in each considered couple of alternatively spliced sequences between which an alternative splicing event is defined (Figure 7b). The output from CLUSTALW (an ALN file) defines the annotation as follows: (residue shared between the two spliced forms); 1 (residue present only in the analyzed splicing pattern); 2 (start and finish position of a gap for the analyzed isoform); 3 (mismatch between the two spliced forms, with substitution between two residues which are very alike; i.e. when the value between the two mismatched residues in the BLOSUM62 matrix [16] is > 0); 4 (mismatch between the two spliced forms, with substitution between two residues which have a value = 0 between them in the BLOSUM62 matrix); 5 (mismatch between the two spliced forms, with substitution between two residues which have opposite properties, e.g. a positively charged and a non-polar residue; i.e. when the value between the two mismatched residues in the BLOSUM62 matrix is < 0) (Figure 7c). It is relevant to understand whether a mismatch is between similar residues (a high value in the BLOSUM62 matrix) because the properties of similar residues, such as polarity or dimensions, are alike; thus a substitution between such residues would not affect the whole protein structure.

- FeatureMap3D [15] is a Web tool which permits to blast a protein sequence against PDB in order to search for the protein structure in PDB (BLASTP is used to align the query sequence against PDB). If an annotation is added to the blasted sequence (by submitting a TAB file instead of a FASTA file), FeatureMap3D differently colors the differently annotated parts of the retrieved structure; otherwise it uses the alignment of the query sequence against the PDB structure as annotation for the different colors and generates a PyMOL script and an annotated pairwise alignment. From FeatureMap3D different types of outputs may be downloaded: the PDB file of the identified structure; the PyMOL script to color the structure as defined by the annotation; the PNG image of the structure; the FeatureMap3D report, which is divided in two parts: first, a summary of the information about the protein (protein name, user's defined name, PDB identifier, structure's resolution, and a report of the BLAST between the query protein sequence and PDB), and second, a table of the protein structural properties, residue by residue (Figure 7d). The report file is used to extract all the structural information imported and stored into the PASS database.

## Competing interests

The authors declare that they have no competing interests.

## Authors' contributions

MD developed and tested all databases and software modules of the PASS Web application and wrote this manuscript. MM was responsible for the supervision and coordination of the project, was involved in the design of the PASS databases and Web application, and contributed to write this manuscript.
